# Core and bridging symptoms in patients with atrial fibrillation: a network analysis

**DOI:** 10.3389/fcvm.2025.1617872

**Published:** 2025-11-07

**Authors:** Dingce Sun, Xue Yang, Hong Li, Guirong Li, Hairong Lin

**Affiliations:** 1 Department of Urology, Mianyang Central Hospital, School of Medicine, University of Electronic Science and Technology of China, Mianyang, China; 2 School of Nursing, Chengdu Medical College, Chengdu, China; 3 Department of Gastroenterology, Mianyang Central Hospital, School of Medicine, University of Electronic Science and Technology of China, Mianyang, China; 4 Department of Nursing, Mianyang Central Hospital; School of Medicine, University of Electronic Science and Technology of China, Mianyang, China

**Keywords:** atrial fibrillation, symptom network, symptom cluster, frail, mental health

## Abstract

**Background:**

Atrial fibrillation symptoms are diverse and complex, but symptom networks can visually map the relationships between symptoms and influencing factors, identifying key symptoms and offering better targets for symptom management. However, research on establishing symptom networks in Atrial fibrillation patients is limited.

**Aim:**

We aimed to construct a symptom network for patients with atrial fibrillation, understand its characteristics, and identify core and bridging symptoms.

**Methods:**

This cross-sectional study enrolled 384 patients with atrial fibrillation from November 2021 to August 2022 at Tianjin Medical University General Hospital of China. Network analysis methods were utilized to construct the symptom network. Centrality metrics were used to identify important symptoms.

**Results:**

By incorporating covariates into the symptom network, we revealed that the Mental Health Inventory-5 score was most closely related to “fatigue at rest”. Sex influenced all symptoms except “dizziness” and “shortness of breath at rest”. Left ventricular ejection fraction was closely connected to “exercise intolerance” and “shortness of breath at rest”, while the frail score was closely linked to “exercise intolerance” and “dizziness”. Controlling for covariates, “shortness of breath during physical activity” and “shortness of breath at rest” are atrial fibrillation patients' core symptoms. “Shortness of breath at rest”, “palpitations”, and “chest pain” served as bridging symptoms between symptom clusters.

**Conclusion:**

Symptom networks can help us understand the relationships between symptoms and influencing factors, as well as the interactions between different atrial fibrillation symptoms.

## 1 Introduction

Atrial fibrillation (AF) is a common cardiac arrhythmia (1) affecting about 1.6% of Chinese adults (2). Atrial fibrillation is associated with increased morbidity, mortality, and a significant economic burden (1). The development of AF is influenced by multiple factors. Conditions such as hyperthyroidism (3) and hypertrophic cardiomyopathy (4) are known to predispose individuals to AF. Additionally, modifiable lifestyle factors are significantly associated with AF risk. Research indicates that smoking more than doubles the risk of AF (5), while endurance exercise training may increase the probability of developing AF by 2- to 10-fold (6). Furthermore, cardiac channelopathies are also closely linked to the occurrence of arrhythmias (7). Further, many atrial fibrillation patients experience symptoms like fatigue, shortness of breath, palpitations (8),, leading to emotional distress and poor quality of life (9, 10). Atrial fibrillation symptoms were strongly associated with multiple factors such as depression, sex, coronary artery disease, diabetes mellitus, and sleep disturbances (11, 12). However, it remains unclear how these factors influence individual or multiple symptoms of atrial fibrillation.

Network analysis is a new approach that offers a comprehensive view by visually representing and quantifying the complex connections between variables in a network graph (13). This method can help visualize the relationships between variables and symptoms, as well as the interactions between different symptoms (14). For example, Bard and colleagues used this method to explore the detailed relationship between insomnia and anxiety or depression symptoms (15). Henneghan and colleagues applied it to examine the detailed connections between symptoms in breast cancer survivors and pro-inflammatory and anti-inflammatory cytokines, suggesting interleukin-2 as a potential mechanism for symptom co-occurrence (16). These examples illustrate that this method can help clarify the relationship between factors and symptoms, aiding in the discovery of symptom mechanisms.

In addition, symptoms of atrial fibrillation are interconnected and mutually influence each other. Different symptoms also play distinct roles and functions within the symptom network (14, 17). Network analysis can help identify core symptoms and bridge symptoms linked to symptom clusters, aiding in understanding the key symptoms driving symptom occurrence and impact, and providing targets for tailored interventions (13). Studies on symptom networks in atrial fibrillation patients are limited; we aim to visualize and analyze the relationships between factors like sleep quality, physiological indicators, psychological health status, and atrial fibrillation symptoms using network analysis. We further seek to explore important symptoms to help understand the mechanisms of symptom interaction to pinpoint intervention targets.

## 2 Design, materials, and methods

### 2.1 Participants and methods

This cross-sectional study enrolled 384 patients with atrial fibrillation from November 2021 to August 2022 via convenient sampling at Tianjin Medical University General Hospital. Patients diagnosed with non-valvular atrial fibrillation (18) were included. Patients with active tumors and reversible atrial fibrillation associated with hyperthyroidism or electrolyte imbalances were excluded.

### 2.2 Sample size

When the network structure contains fewer than 20 nodes, sample sizes ranging from 250 to 350 are generally sufficient to observe moderate sensitivity, high specificity, and strong correlations between edge weights (19). Ultimately, we included 360 participants, meeting the required sample size.

### 2.3 Data collection

#### 2.3.1 Demographics and clinical data

Socio-demographic data such as age, sex, and body mass index; disease details like atrial fibrillation type and atrial fibrillation duration; laboratory indicators including high-sensitivity C-reactive protein and B-type natriuretic peptide; echocardiographic measurements including left atrial anteroposterior diameter, right atrial transverse diameter, left ventricular end-diastolic diameter, right ventricular transverse diameter, and left ventricular ejection fraction. Echocardiographic and laboratory data were collected on the admission day.

#### 2.3.2 Symptom, mental health status, sleep quality, and frailty evaluation tools

Symptoms were evaluated using the University of Toronto Atrial Fibrillation Severity Scale (AFSS), which assesses symptoms such as palpitations, shortness of breath, fatigue, dizziness, and chest pain at rest and during activity. Each item is scored from 0 to 5, with 0 meaning “no symptoms” and 5 meaning “always present.” The total score ranges from 0 to 35. The Cronbach's *α* coefficient for this scale in our study was 0.74 (20).

Mental health was assessed using the Mental Health Inventory-5 (MHI-5), which measures emotional well-being, including anxiety, depression, vitality, happiness, and tranquility over the past month. Scores are transformed to a scale of 0 to 100, with higher scores reflecting better mental health. The Cronbach's *α* coefficient ranged from 0.72 to 0.88 (21).

The Pittsburgh Sleep Quality Index (PSQI) was used to evaluate sleep quality over the past month, with scores ranging from 0 to 21. Higher scores indicate poorer sleep quality. The Chinese version of the PSQI had a Cronbach's *α* coefficient of 0.71 (22).

Frailty was measured using the Chinese version of the FRAIL scale, which assesses fatigue, resistance, ambulation, illness, and weight changes. Each item is scored from 0 to 1, and a total score of 3 or higher indicates frailty. This scale demonstrated good reliability with a Cronbach's *α* coefficient of 0.826 (23).

#### 2.3.3 Data collection methods

Upon admission, informed consent was obtained, and a thorough assessment was conducted, covering sociodemographic, clinical, symptom, sleep quality, and mental health information. Patients were fully informed about the study's goals and guidelines. To reduce bias, survey questions were worded consistently and impartially. Of the 400 questionnaires distributed, 384 valid responses were returned, after excluding incomplete forms or those completed in under 5 min, resulting in a 96% response rate.

This study received approval from the Ethics Committee for Clinical Research at Tianjin Medical University General Hospital [Approval No. (IRB2023-WZ-111)]. All procedures adhered to ethical standards and complied with the Declaration of Helsinki.

### 2.4 Statistical analysis

Statistical analysis utilized SPSS 19.0 and R 4.1.3 software. Missing values for physiological and laboratory data (less than 4% of the sample) were addressed by replacing them with the mean or median. Continuous variables were expressed as mean ± standard deviation or median, while categorical variables were presented as frequencies and percentages. For assessing factors influencing symptom burden in atrial fibrillation patients, we utilized bivariate analysis and linear regression.

#### 2.4.1 Symptom network visualization

Two symptom networks were constructed using distinct graphical approaches: a Mixed Graphical Model (MGM) enrolling covariates which incorporated both continuous (clinical symptoms、left ventricular ejection fraction、frail score、MHI-5 score) and categorical variables (sex), while the “Gaussian Graph” model focused only on continuous variables after controlling for covariates. Covariates were controlled by performing regression analysis on the seven symptom variables, with the residuals from this analysis used as the data for further analysis (24).

The “*qgraph*” package was used for network visualization. The “Fruchterman-Reingold” algorithm positioned highly connected and numerous symptom nodes at the center of the network and less connected and fewer symptom nodes at the periphery. The “pcor” algorithm was used to reduce false positive results in the symptom network visualization. The accuracy and stability of the network were assessed using the “bootnet” package in R. Accuracy was evaluated by calculating 95% confidence intervals for edge weights based on 1,000 nonparametric bootstrap samples. The stability of node centrality was assessed via 1,000 case-dropping bootstrap samples, which were used to compute the correlation stability coefficient (CS).

#### 2.4.2 Core and bridge symptoms identification

We used three centrality measures: “Betweenness,” “Closeness,” and “Strength” to identify key symptoms. “Betweenness” counts how often a node lies on the shortest path between other nodes. “Closeness” calculates the average distance from a node to all others, highlighting influential nodes. “Strength” measures network connectivity, with higher values indicating more frequent co-occurrence. High centrality nodes were identified as core symptoms. Symptom clusters were identified with the “EGAnet” package's walktrap algorithm, and “bridge strength” was used to find symptoms linking clusters. The “walktrap” algorithm is a built-in function of the “EGAnet” package in R software. It works by calculating the distances between nodes in a graph to identify community structures.

## 3 Results

### 3.1 Participant characteristics

The average age of participants was 66.19 ± 9.38 years, with 54.7% male. Of the participants, 58.3% had paroxysmal atrial fibrillation, and the median duration was 18 months (IQR: 7–65). Hypertension and coronary heart disease were present in 62% and 36.5% of participants, respectively (Table 1). The average PSQI and MHI-5 scores were 7.85 and 74.64, respectively, with 61.9% of participants experiencing impaired sleep quality (PSQI > 5).

**Table 1:** Demographic and clinical characteristics of patients with atrial fibrillation (*N* = 384).

Item	*`x* *±* *S/n(%)/M(IQR)*
Age (year)	66.19 ± 9.38
Sex (male)	210 (54.7%)
BMI (Kg/m^2)^	25.68 ± 3.41
AF Duration (month)	18 (7,65)
paroxysmal AF	224 (58.3%)
Diabetes	75 (19.5%)
Hypertension	238 (62%)
Coronary heart disease	140 (36.5%)
Ischemic stroke	91 (23.7%)
COPD	7 (1.8%)
OSA	10 (2.6%)
Heart failure	46 (12%)
Medication history	
None	227 (59.3%)
AADs	65 (17%)
Rate control	76 (19.6%)
AADs + rate control	16 (4.1%)
RFCA history	75 (19.6%)
Hs-CRP (mg/L)	1.41（0.70, 3.46）
BNP (pg/ml)	138 (65, 276）
LA-ap (mm)	42.07 ± 5.70
LVEDD (mm)	48.25 ± 4.42
RA-t (mm)	40.08 ± 5.21
RV-b (mm)	32.17 ± 3.42
LVEF (%)	62%（60%, 64%）
MHI-5 score	74.64 ± 16.31
PSQI score	7.85 ± 4.33
Frail score	1.47 ± 1.30

M, median; IQR, interquartile range; BMI, body mass index; RFCA, radiofrequency catheter ablation; AF, atrial fibrillation; BNP, B-type natriuretic peptide; LVEF, left ventricular ejection fraction; RA-t, right atrial transverse diameter; LA-ap, left atrial anteroposterior diameter; LVEDD, left ventricular end-diastolic diameter; RV-b, right ventricular transverse diameter; Hs-CRP, high-sensitivity C-reactive protein; AADs, anti-arrhythmic drugs; OSA, obstructive sleep apnea; COPD, chronic obstructive pulmonary disease; MHI-5, Mental Health Inventory-5; PSQI, The Pittsburgh Sleep Quality Index.

### 3.2 Analysis of the current status and influencing factors of symptom burden in patients with atrial fibrillation

The AFSS score was 9.28 ± 5.19, with 24 patients (6.3%) reporting no symptoms. A total of 360 patients were included in the analysis after excluding non-symptom patients. The bivariate analysis revealed statistical differences in sex, coronary heart disease, heart failure, chronic obstructive pulmonary disease, and atrial fibrillation classification. Variables such as a B-type natriuretic peptide, left ventricular ejection fraction(LVEF), sleep quality, frailty, mental health status, high-sensitivity C-reactive protein, and left atrial diameter showed a correlation with symptom burden (*p* < 0.05), as detailed in Table 2. Multiple linear regression indicated that sex (*β* = 18.8, *p* = 0.007), MHI-5 score (*β* = −0.06, *p* = 0.001), left ventricular ejection fraction (*β* = −13.56, *p* = 0.010), and frail score (*β* = 0.68, *p* = 0.005) were identified as independent influencing factors of symptom burden (Table 3).

**Table 2:** The bivariate analysis of symptom burden in atrial fibrillation patients (*N* = 360).

Item	*t/F/r*	*p*
Age (year)	0.05	0.322
Sex (male)	−3.84	<0.001*
BMI (Kg/m2)	−0.04	0.427
AF Duration (month)	0.07	0.209
paroxysmal AF	−1.95	0.051
Diabetes	0.17	0.867
Hypertension	−0.53	0.595
Coronary heart disease	−2.91	0.004*
Ischemic stroke	0.57	0.572
COPD	−2.44	0.015*
OSA	−0.19	0.848
Heart failure	−2.89	0.004*
Medication history	0.46	0.708
None		
AADs		
Rate control		
AADs + rate control		
RFCA history	−1.59	0.113
Hs-CRP (mg/L)	0.16	0.002*
BNP (pg/ml)	0.17	0.001*
LA-ap (mm)	0.08	0.138
LVEDD (mm)	0.05	0.305
RA-t (mm)	0.01	0.882
RV-b (mm)	0.00	0.999
LVEF (%)	−0.19	<0.001*
MHI-5 score	−0.28	<0.001*
PSQI score	0.20	<0.001*
Frail score	0.33	<0.001*

BMI, body mass index; RFCA, radiofrequency catheter ablatsion; AF, atrial fibrillation; BNP, B-type natriuretic peptide; LVEF, left ventricular ejection fraction; RA-t, right atrial transverse diameter; LA-ap, left atrial anteroposterior diameter; LVEDD, left ventricular end-diastolic diameter; RV-b, right ventricular transverse diameter; Hs-CRP, high-sensitivity C-reactive protein; AADs, anti-arrhythmic drugs; OSA, obstructive sleep apnea; COPD, chronic obstructive pulmonary disease; MHI-5:Mental Health Inventory-5; PSQI, The Pittsburgh Sleep Quality Index.

*p < 0.05.

**Table 3:** Multiple linear regression for symptom burden of atrial fibrillation patients (*N* = 360).

Characteristic	*β*	*SE*	*β’*	*t*	*p*
Common	18.80	4.59		4.09	0.000
Sex (female)	1.51	0.56	0.14	2.70	0.007*
LVEF (%)	−13.56	5.25	−0.15	−2.58	0.010*
MHI-5 score	−0.06	0.02	−0.17	−3.29	0.001*
Frail score	0.68	0.24	0.17	2.83	0.005*
Heart failure	0.72	0.87	0.05	0.83	0.406
COPD	2.52	1.86	0.07	1.35	0.177
Coronary heart disease	0.56	0.60	0.05	0.94	0.351
AF type	0.53	0.60	0.05	0.88	0.380
BNP (pg/ml)	0.00	0.00	0.03	0.44	0.663
PSQI score	0.09	0.07	0.07	1.28	0.201
Hs-CRP (mg/L)	0.00	0.04	−0.01	−0.10	0.923
LA-ap (mm)	0.00	0.06	0.00	−0.03	0.973

R^2^ = 21.3%, adjusted R^2^ = 18.4%, F = 7.344, P < 0.001.

AF, atrial fibrillation; BNP, B-type natriuretic peptide; LVEF, left ventricular ejection fraction; LA-ap, left atrial anteroposterior diameter; Hs-CRP, high-sensitivity C-reactive protein; COPD, chronic obstructive pulmonary disease; PSQI, The Pittsburgh Sleep Quality Index.

*p < 0.05.

### 3.3 Symptom network of atrial fibrillation patients after incorporating covariates

**Figure 1:**
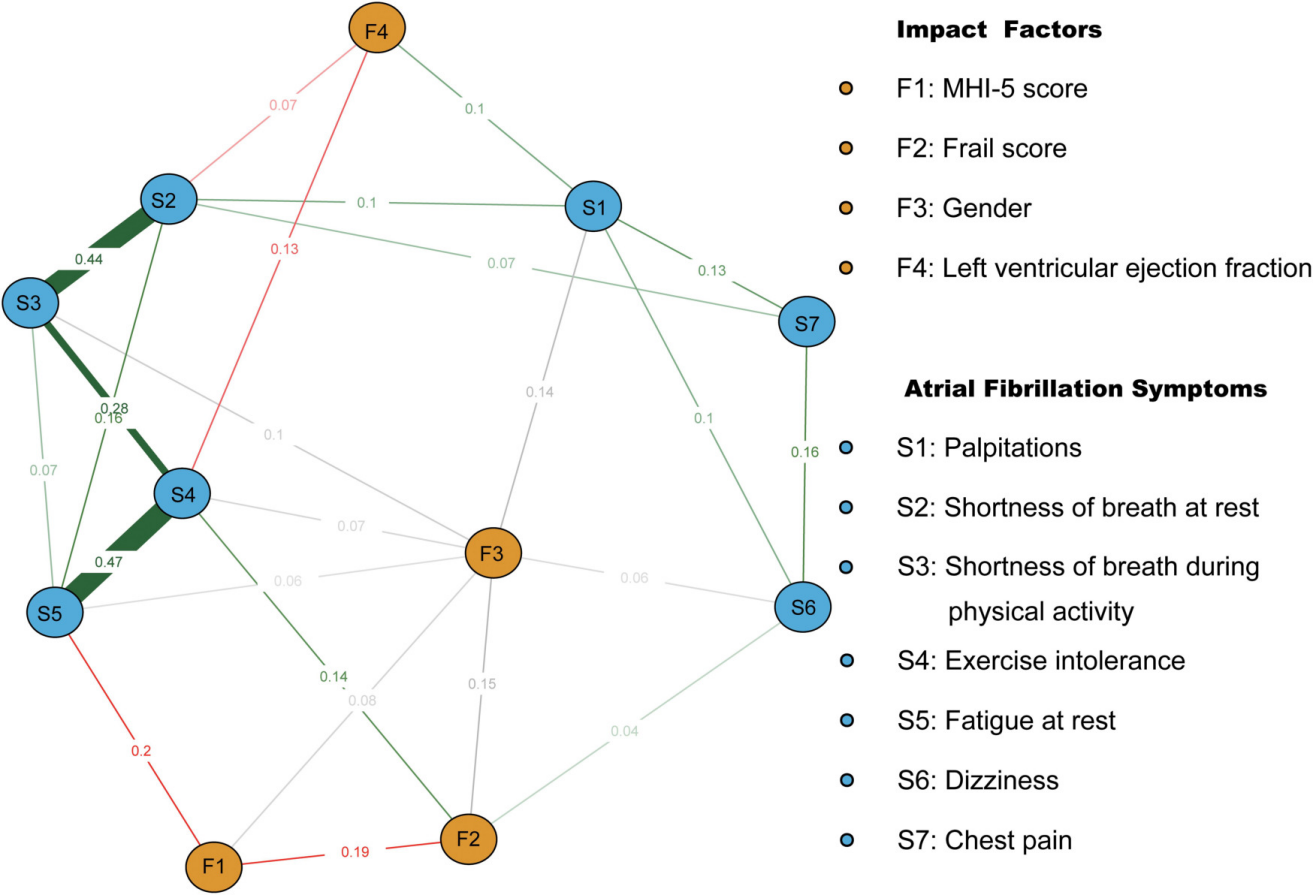
Symptom network of atrial fibrillation patients after incorporating covariates. Red and green line segments represent the association between continuity variables, with red line segments representing a negative correlation between nodes and green representing a positive correlation between nodes. Gray line segments indicate the relationship between categorical covariates and other nodes. Wider segments indicate stronger connectivity between the two.

Symptoms and codes are named in Table 4. After incorporating covariates such as MHI-5 score, sex, left ventricular ejection fraction, and frail score into the symptom network, it was observed that the MHI-5 score was most closely related to S5 (edge weight = 0.2). Sex influenced all symptoms except S7 and S2. left ventricular ejection fraction was closely connected to S4 (edge weight = 0.13) and S2 (edge weight = 0.07). Frail score was closely linked to S4 (edge weight = 0.14) and S6 (edge weight = 0.04). There were also significant correlations between frail score, MHI-5 score, and sex (Figure 1).

**Table 4:** Symptom code.

Symptoms	*Item*
Palpitation	S1
Shortness of breath at rest	S2
Shortness of breath during physical activity	S3
Exercise intolerance	S4
Fatigue at rest	S5
Dizziness	S6
Chest pain	S7

### 3.4 The symptom network of atrial fibrillation patients after controlling for the covariates

**Figure 2:**
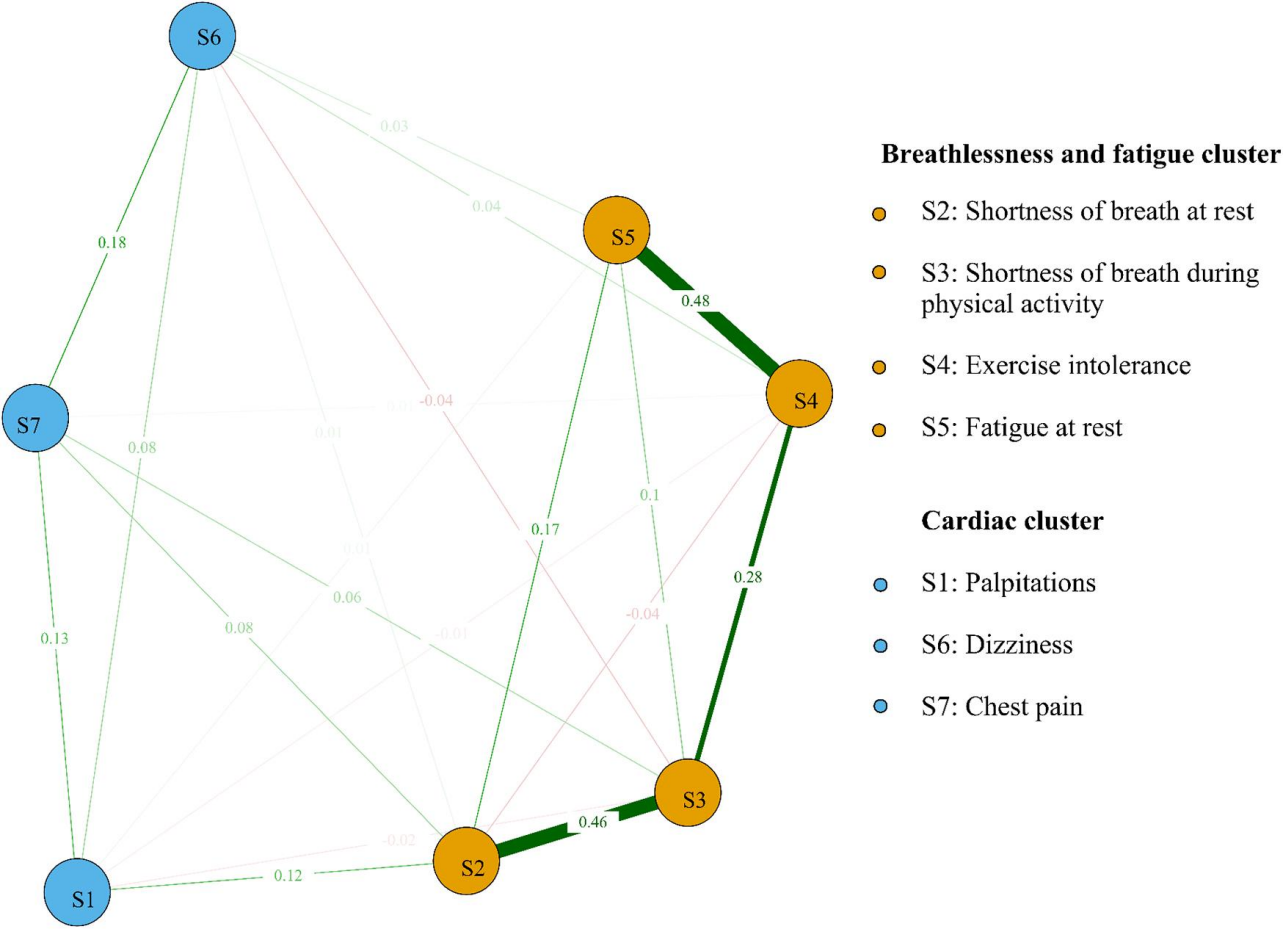
Symptom network and clusters of atrial fibrillation patients. Red line segments represent a negative correlation between nodes, and green represents a positive correlation between nodes. Wider segments indicate stronger connectivity between the two nodes.

**Figure 3:**
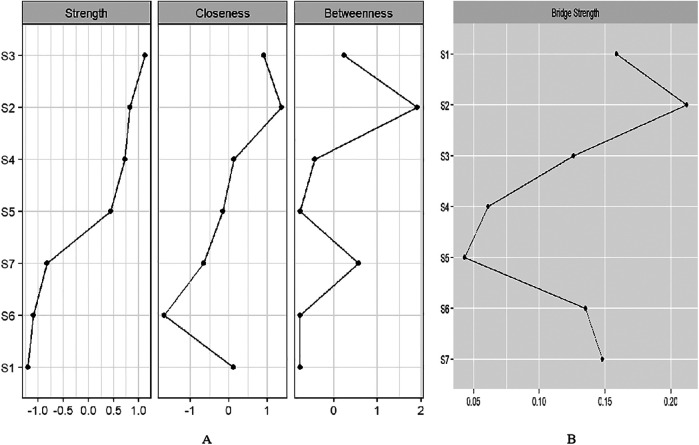
**(A)** Centrality indices of “Strength”, “Closeness”, and “Betweenness” for 7 symptoms ordered by “Strength”. **(B)** Bridge strength of symptom nodes.

We identified the top three symptom connections in the network: S4 with S5 (edge weight  = 0.48), S2 with S3 (edge weight = 0.46), and S3 with S4 (edge weight = 0.28). Most symptoms showed positive correlations, except for the negative correlations between S2 and S4, S3 and S6, and S1 and S4 (Figure 2). The three strongest “Strength” indicators were S3 (node strength = 0.96), S2 (node strength = 0.88), and S4 (node strength = 0.86). This suggests that the core symptoms of atrial fibrillation patients are shortness of breath during physical activity and at rest (Figure 3A).

We identified two symptom clusters: “cardiac cluster” (S1, S6, S7) and “breathlessness and fatigue cluster” (S2, S3, S4, S5) (Figure 2). The top three symptoms with the highest bridge strength were S2 (node bridge strength = 0.21), S1 (node bridge strength = 0.16), and S7 (node bridge strength = 0.15), indicating their role in linking the clusters (Figure 3B).

### 3.5 Accuracy and stability of the symptom network

**Figure 4:**
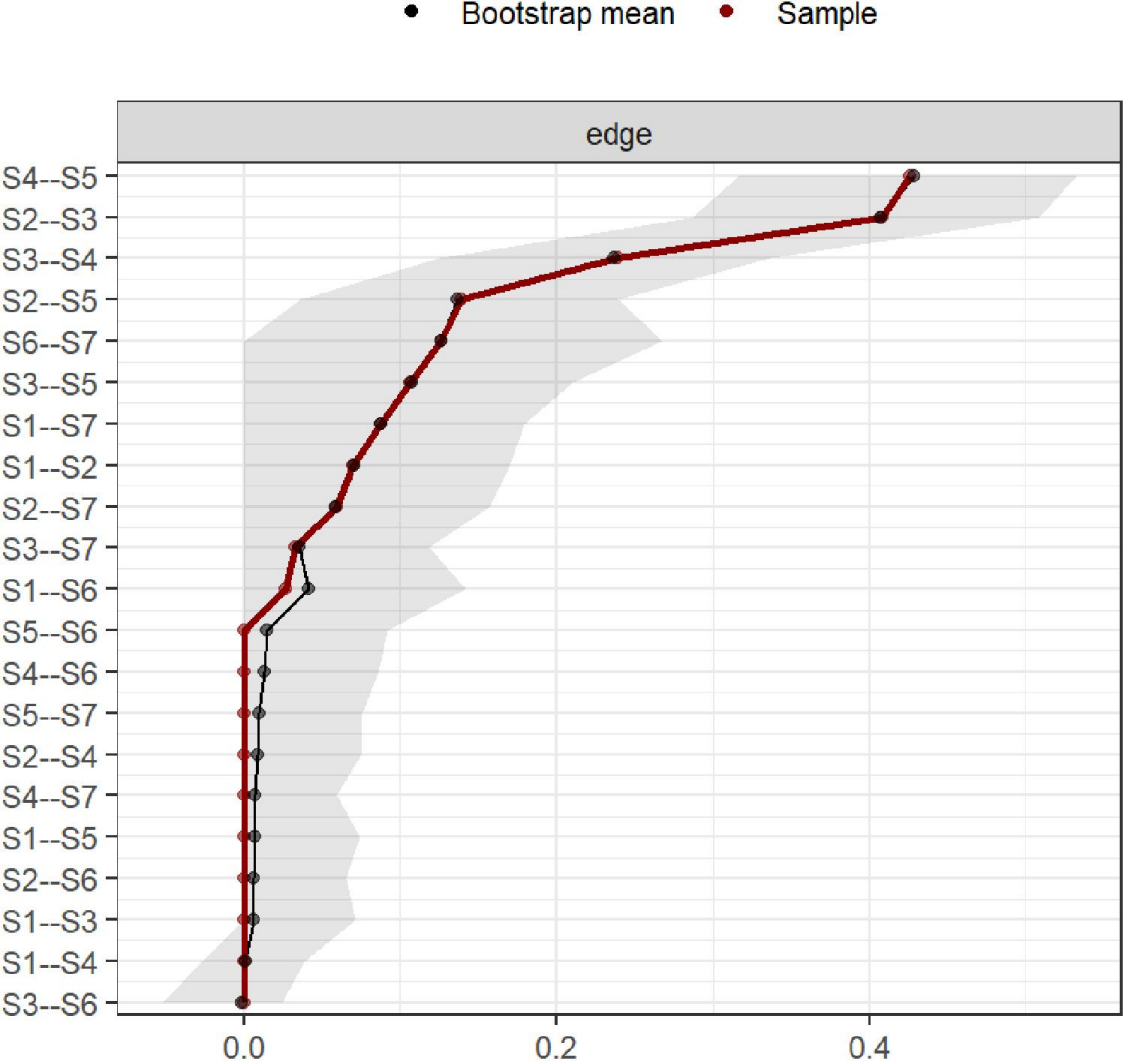
Bootstrap analysis results of edge weights.

**Figure 5:**
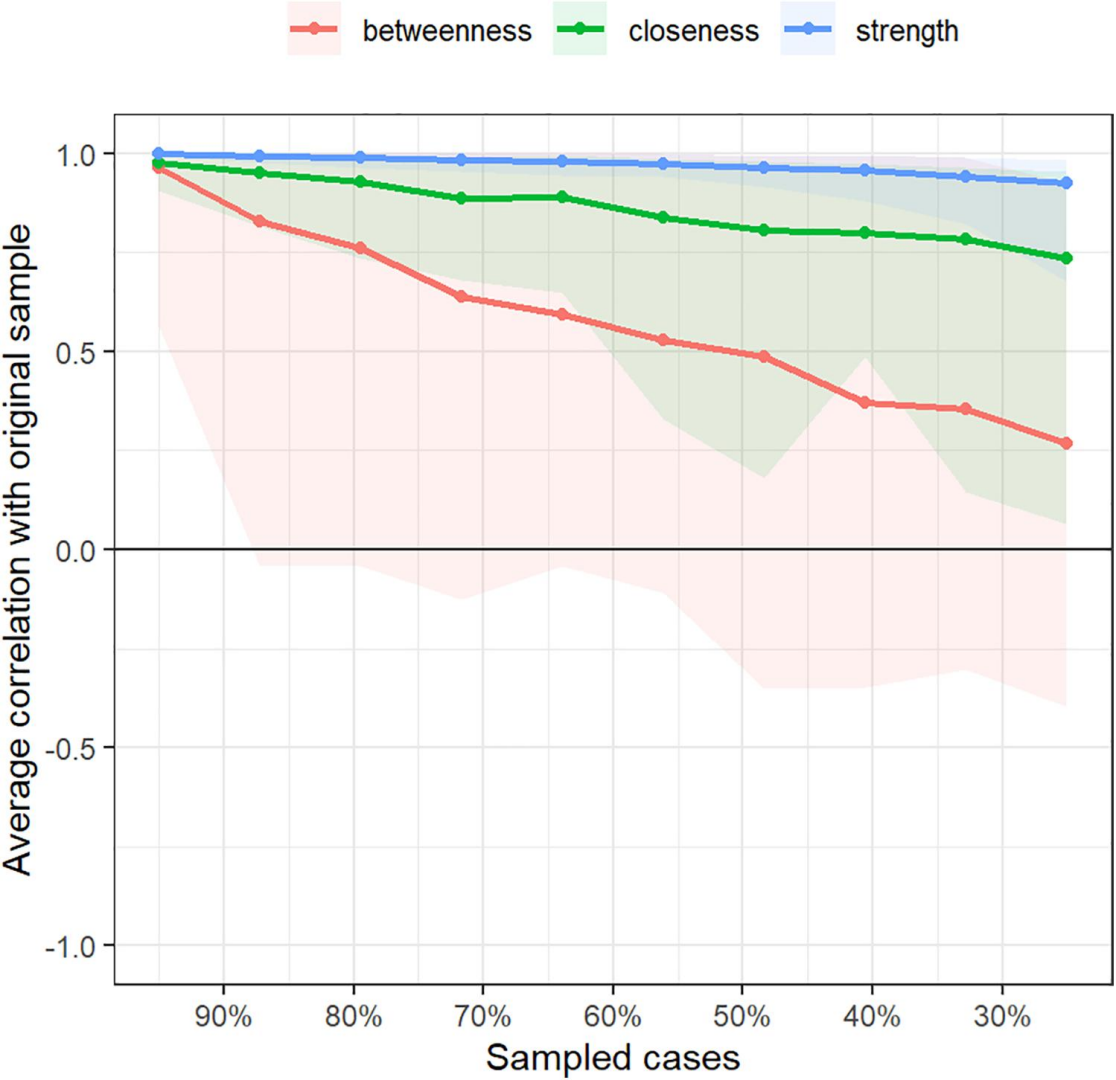
Correlation stability coefficient for strength, betweenness, and closeness of symptom network.

Bootstrap confidence intervals for edge weights were relatively narrow, indicating good accuracy of the symptom network (Figure 4). In this study, the CS values for symptom network “strength” centrality were 0.75. These values indicate good stability for symptom node centrality (Figure 5).

## 4 Discussion

We observed that worsening mental health status was linked to a higher symptom burden, which aligns with previous research (25). Negative emotions were found to be strong predictors of symptom severity in atrial fibrillation patients (26), affecting symptom perception through attentional bias (27) and psychological rumination (28), often leading to an overestimation of symptom frequency and severity. The study also found a strong association between mental health status and Shortness of breath at rest, similar to Yu (29). This may be related to the chronic low-grade inflammation in atrial fibrillation, which sensitizes the amygdala circuit, activates the neuroimmune network, and induces autonomic hyperreactivity, resulting in difficulty breathing and intolerance to exercise (30). Therefore, healthcare professionals should recognize the impact of emotional distress on symptomatology in atrial fibrillation patients and provide proactive psychological support.

Female patients experience a more severe symptom burden compared to males, a phenomenon supported by numerous studies (31). This may be due to them less likely to be treated with rhythm control strategies (32), more extensive low-voltage areas in the left atrium, higher rates of complex fractionated atrial electrograms (33), and more severe atrial fibrosis in females (34), leading to more pronounced atrial remodeling and increased symptom burden. Furthermore, the study finds that sex has a broad impact on atrial fibrillation symptoms, although the mechanisms remain unclear and require further exploration.

Left ventricular ejection fraction is closely associated with symptoms of exercise intolerance and shortness of breath at rest. The left ventricular ejection fraction reflects the heart's ability to pump blood and its overall function. During atrial fibrillation episodes, hemodynamic changes lead to inadequate blood ejection, causing symptoms like dyspnea and exercise intolerance (35–39). In addition, lower left ventricular ejection fraction is closely linked to persistent atrial fibrillation which experiences more pronounced symptoms of fatigue and exercise intolerance (40).

The more severe the frailty, the heavier the symptom burden, similar to findings in the Slawuta study (41). Frailty, caused by factors such as metabolic and neuroimmune dysfunction, may contribute to increased symptom burden by promoting atrial remodeling through chronic inflammatory responses (42). Covariate-controlled symptom networks show a closer association between frailty and exercise intolerance, as well as chest pain. One possible reason is the similarity between symptoms of atrial fibrillation and frailty, with impaired physical activity and mobility being key features of frailty. Moreover, both conditions share pathological mechanisms such as inflammation and oxidative stress (43). Frailty is also often characterized by a decline in skeletal muscle quantity and quality, which may contribute to exercise intolerance (42). Additionally, covariate-controlled symptom networks indicate a strong association between frailty and mental health status, as well as sex, highlighting the need for multidisciplinary interventions addressing various aspects such as sex and psychological factors to effectively manage symptoms in atrial fibrillation patients with frailty and improve the symptom network.

After controlling for confounding factors and covariates, the network shows that palpitations are negatively correlated with exercise intolerance, while dizziness and exercise intolerance are negatively correlated with shortness of breath. This may be due to atrial fibrillation reducing cardiac output (35–39), leading to symptoms such as exercise intolerance and dizziness. At the same time, reduced cardiac output may decrease pulmonary circulation congestion, which could, in turn, alleviate shortness of breath at rest.

We found that the core symptoms in atrial fibrillation patients were shortness of breath during physical activity and at rest. This is consistent with our previous study (17), highlighting the stability of core symptoms and their representative role in symptomatology. Core symptoms can trigger a range of connected symptoms that can signal the start or worsening of other issues (14). During atrial fibrillation episodes, irregular atrial contractions and reduced ventricular diastolic time decrease effective cardiac output, causing compensatory shortness of breath (35, 36). Hemodynamic changes may increase left ventricular filling pressure, contributing to exercise intolerance (37). Furthermore, atrial fibrillation patients often have endothelial dysfunction and impaired peripheral muscle oxygen uptake, which, along with hemodynamic changes, can result in fatigue and weakness due to altered muscle sensing (38, 39). We identified two symptom clusters involving “cardiac cluster” and “breathlessness and fatigue cluster” in patients with atrial fibrillation, similar to Streur (44). “Shortness of breath at rest”, “palpitations” and “chest pain” were a bridge of clusters with high “bridge strength”. These symptoms can be targeted to improve overall symptom management (14). Breathing difficulties can worsen chest pain by affecting the sympathetic nervous system (37), triggering a cardiac symptom cluster. Additionally, palpitations and chest pain may decrease the desire for physical activity in atrial fibrillation patients, potentially leading to long-term muscle changes and exercise intolerance.

Our study suggests that focusing solely on controlling heart rate and rhythm in atrial fibrillation patients may not be enough (1). Paying attention to core symptoms like shortness of breath could help improve the entire symptom network. Although evidence is limited, treatments such as an ablation procedure and moderate exercise may help ease core symptoms and improve overall symptom management (35, 45).

### 4.1 Strengths and limitations

Using network analysis, we identified the mechanisms linking symptoms and influencing factors. By calculating centrality measures and bridge strength, we were able to pinpoint core and bridging symptoms, which helps in understanding the emergence of symptoms. This approach provides a better understanding of atrial fibrillation symptoms. However, this study has several limitations: it was a single-center investigation with a small sample size, limiting generalizability; its cross-sectional design reduces the strength of evidence regarding relationships between atrial fibrillation symptoms and other factors; and the use of convenience sampling may have introduced selection bias. Longitudinal studies would better elucidate symptom progression and causal relationships with influencing factors.

## 5 Conclusion

Employing symptom networks helps uncover the underlying mechanisms behind symptom occurrence, providing a clearer path for identifying targets for symptom management. Mental health was most closely related to “fatigue at rest”. Sex influenced all symptoms except “dizziness” and “shortness of breath at rest”. Left ventricular ejection fraction was closely connected to “exercise intolerance” and “shortness of breath at rest”, while the frail score was closely linked to “exercise intolerance” and “dizziness”. Shortness of breath during physical activity and at rest are identified as core symptoms, while “shortness of breath at rest”, “palpations” and “chest pain” serve as bridging symptoms.

## Data Availability

The raw data supporting the conclusions of this article will be made available by the authors, without undue reservation.
